# Energy drink consumption among Australian adolescents associated with a cluster of unhealthy dietary behaviours and short sleep duration

**DOI:** 10.1186/s12937-021-00719-z

**Published:** 2021-07-05

**Authors:** Tegan Nuss, Belinda Morley, Maree Scully, Melanie Wakefield

**Affiliations:** 1 Centre for Behavioural Research in Cancer, Cancer Council Victoria, 615 St Kilda Road, Melbourne, VIC 3004 Australia; 2 Melbourne School of Psychological Sciences, The University of Melbourne, Parkville, VIC 3010 Australia

**Keywords:** Energy drinks, Adolescents, Health behaviours, Australia

## Abstract

**Background:**

Non-alcoholic energy drinks (‘energy drinks’) are high in sugar, as well as caffeine, leading to concerns regarding their suitability for children and adolescents. Despite this, marketing of energy drinks is often directed at adolescents, and there are no age restrictions on the sale of these products in Australia. The current study aimed to examine patterns in consumption of energy drinks among Australian secondary school students and identify sociodemographic and behavioural correlates associated with regular consumption.

**Methods:**

Participants were 8942 students in Years 8 to 11 (aged 12 to 17 years) who participated in the 2018 National Secondary Students’ Diet and Activity (NaSSDA) cross-sectional survey. A multistage stratified random sampling procedure was used. Within the school setting, students self-completed an online questionnaire assessing their dietary, physical activity and sedentary behaviours. A multilevel logistic regression model was used to examine associations between energy drink consumption and sociodemographic and behavioural factors.

**Results:**

Overall, 8% of students reported consuming energy drinks on a weekly basis (‘regular consumers’). A further 16% indicated they consume less than one cup per week of these types of drinks, while around three-quarters (76%) reported they do not consume energy drinks. Regular consumption of energy drinks was independently associated with being male, having greater weekly spending money, high intakes of snack foods, fast food, other sugar-sweetened beverages and fruit juice, as well as short sleep duration. There was no independent association with other sociodemographic characteristics (i.e., year level, level of disadvantage, geographic location), consumption of vegetables and fruit, physical activity level, or sedentary recreational screen time.

**Conclusions:**

While most Australian adolescents do not consume energy drinks, regular consumption is more prevalent among males, and consumption appears to cluster with other unhealthy dietary behaviours and short sleep duration. Findings support the need for policies that will reach identified at-risk groups (e.g., increased regulation of the marketing and sale of energy drinks), as well as suggest opportunities for interventions targeting energy drink consumption alongside other unhealthy dietary behaviours.

## Introduction

Population monitoring studies show that rates of overweight and obesity are at concerning levels globally [1]. Given the impact of overweight and obesity on rates of noncommunicable disease [2] and costs to healthcare systems and productivity [3, 4], there is an urgent need for public health programs and policies to help increase healthy eating and reduce sedentary behaviour. Sugary drinks are energy-dense and offer no nutritional value, and as a result have been identified as an effective target for obesity prevention [5]. While consumption of sugary drinks broadly may be decreasing [6–8], there is some evidence that consumption of specific categories of sugary drinks, such as non-alcoholic energy drinks (‘energy drinks’), may be rising [7, 9]. Volume sales of energy drinks increased markedly over the last two decades in Australia, with an annual growth rate of 170% estimated between 1997 and 2018 [10, 11]. Data from Australia’s National Nutrition and Physical Activity Survey (NNPAS) 2011–12 show that consumption of energy, electrolyte and fortified drinks on the day prior to interview was highest among adolescents and young adults (14–30 years) [8].

In Australia, the volume of energy drinks sold range from 250 to 550 ml [12], with a 500 ml serving size containing up to 21 teaspoons or 83.5 g of sugar [13], as well as the equivalent caffeine content of two cups of instant coffee (i.e., 160 mg caffeine) [14]. Consumption of a 500 ml energy drink results in sugar intake over three times the daily limit recommended by the World Health Organization [15], which is concerning given excess sugar consumption from sugary drinks can lead to weight gain and obesity [16]. Additionally, due to the high level of caffeine present in energy drinks and the potential harm to young people, the American Academy of Paediatrics has concluded that these beverages are not suitable for consumption by children and adolescents [17].

Despite a voluntary commitment by industry to responsibly sell and promote energy drinks in Australia [18], the targeted promotion of these products to adolescents has been identified as a key driver of consumption among this population [19–21]. Energy drinks are branded with names that appeal to young people (e.g., ‘Rockstar’ and ‘Full Throttle’) and are commonly associated with events enjoyed by young people, such as extreme sports and music festivals [22, 23]. While age-specific restrictions on the sale of energy drinks have been introduced in some European countries (e.g., England, Lithuania, Latvia), these high sugar and caffeine content drinks are readily available for purchase by Australian adolescents in supermarkets, convenience stores, and service stations.

Globally, there has been some research into the factors associated with energy drink consumption in adolescents. In addition to sociodemographic factors, studies have most commonly examined the association between energy drink consumption and risky behaviours, such as substance use [9, 24–39], while a smaller number of studies have investigated the association between energy drink consumption and dietary and lifestyle behaviours [31, 37, 39–43]. For example, a 2016 review reported that energy drink consumption was consistently linked to alcohol use and cigarette smoking, and appeared to be positively related to various other unhealthy behaviours, such as skipping breakfast, consumption of junk food and other sugary drink varieties, and regular video game use [44]. Previous studies have also reported a link between various sleeping difficulties, such as shorter sleeping duration, and consumption of energy drinks [44–46].

To date, little research has examined the factors associated with energy drink consumption among Australian adolescents. In a state-based study, Hardy et al. utilised data from 3671 children aged 10–16 years who participated in the 2015 New South Wales (NSW) Schools Physical Activity and Nutrition Survey (SPANS) to investigate the association between consumption of various sugary drink types and oral health and weight status [47]. Their analyses found that energy drinks were the most commonly consumed of the sugary drink varieties examined and consumers were significantly more likely to be classified as having overweight or obese body mass index (BMI). Further studies are needed to investigate the dietary and lifestyle factors that may be associated with Australian adolescents’ consumption of energy drinks in order to identify target groups and inform public health policies and programs directed at reducing consumption among young people. Accordingly, this study aimed to examine patterns in consumption of energy drinks amongst Australian adolescents and to identify demographic and behavioural correlates associated with regular consumption.

## Method

### Sample and procedure

The sample comprised Australian adolescents who participated in the 2018 National Secondary Students’ Diet and Activity (NaSSDA) survey conducted between May and December 2018. Students were from year levels 8 to 11 (aged 12 to 17 years) drawn from all states and territories and from across the three education sectors (government, Catholic and independent). Sampling was undertaken by the Australian Council for Educational Research (ACER: https://www.acer.org/au/). A multistage stratified random sampling technique was employed with schools proportionally randomly selected from each state and territory at the first stage (104 schools in total), and non-streamed classes (i.e. not formed on the basis of academic ability or interest) randomly selected within schools at the second stage. Where possible, one class group (around 25 students) was selected from each year level. Additional classes were selected where class sizes were small, consent rates were expected to be low, and/or the school did not enrol students in all year levels. Schools that declined to participate were replaced in the sample by a school with comparable characteristics (e.g., education sector, location based on postcode). The study required students to complete an online self-report questionnaire which assessed their dietary and physical activity behaviours using validated assessment tools. The questionnaire was completed in students’ usual class groups. Participation in the study required student and parent/carer consent. Approval for the study was given by the Human Research Ethics Committee of Cancer Council Victoria (approval number HREC 1117), relevant state/territory education authorities and school principals.

### Measures

#### Energy drink consumption

Students’ frequency of consuming energy drinks was assessed using a single-item question: “*How much non-alcoholic energy drinks (like Red Bull, V, Mother) do you usually drink? (1 cup = 250 ml)*”. Pre-coded response options included: ‘I don’t drink non-alcoholic energy drinks’; ‘Less than one cup a month’; ‘About 1–3 cups a month’; ‘Less than 1 cup a week’; ‘About 1–3 cups a week’; ‘About 4–6 cups a week’; ‘About 1–2 cups a day’; ‘About 3–4 cups a day’; ‘5 cups or more a day’. While the reliability and validity of this measure has not been assessed, the question was adapted from those used to assess consumption of other beverage varieties (detailed below) and was similar to a measure used elsewhere to assess energy drink consumption among adolescents and shown to have acceptable test-retest agreement [39]. Consistent with previous studies [9, 39], students who reported consuming energy drinks at least weekly (i.e., ≥1 cup/week) were categorised as ‘regular consumers’.

#### Sociodemographic characteristics

Students were asked to record their sex, school year level, and residential postcode. Residential postcode was used to categorise students into low (first and second quintiles), medium (third and fourth quintiles) and high (fifth quintile) socio-economic groups according to the Australian Bureau of Statistics’ Index of Relative Socio-Economic Disadvantage (IRSD) [48]. This index ranks geographic areas (postcodes) on a continuum from high to low disadvantage taking into consideration characteristics such as education, income, occupation and housing that may reduce socio-economic conditions of the area. Residential postcode was also used to determine geographic location, with students classed as metropolitan or rural/regional according to the Australian Statistical Geography Standard (ASGS) Remoteness Structure [49]. In order to provide an indication of their capacity to make independent purchasing decisions, students were also asked to indicate the amount of money they have available to spend on themselves in a typical week (e.g., from pocket money, part-time job). Response options included: ‘None’; ‘Less than $10 per week’; ‘$10–19 per week’; ‘$20–39 per week’; ‘$40–59 per week’; ‘$60–79 per week’; ‘$80–99 per week’; and ‘$100 per week or more’. Students were categorised as having either less than AUD$10, $AUD$10–39, or AUD$40 or more weekly spending money.

#### Eating behaviours

Students’ intake of key healthy and unhealthy foods were assessed using brief questions adapted from recommendations from the NSW Centre for Public Health Nutrition, Australia [50]. Questions used pre-coded response categories, with similar questions assessing dietary intake previously demonstrated to distinguish categories of intake among 10- to 12-year-olds [51]. Specifically, students were asked to indicate how many daily servings of vegetables (not including potatoes, hot chips, or fried potatoes) and fruit (not including juice), how much soft drinks, sports drinks, and cordials (i.e., sugary drinks) and fruit juice (all varieties) they usually consume, and how often they have meals or snacks (such as burgers, pizza, chicken or chips) from fast food or takeaway food places. To assess consumption of snack foods, students were asked to indicate how often they eat four varieties of snack foods: ice cream, icy poles or ice blocks; potato crisps/chips or other salty snacks; confectionery; and, sweet foods. Weekly snack food consumption was subsequently derived by assigning a weekly equivalent value to each response option and then summing students’ responses for the four snack food varieties. As per previous NaSSDA papers [52–54], variables measuring students’ eating behaviours were binary coded to indicate low intake of healthy foods (≤1 daily serving of vegetables and fruit respectively) and high/frequent consumption of unhealthy foods (≥4 cups/week of sugary drinks and fruit juice respectively; ≥1 time/week fast food; ≥14 times/week snack foods).

#### Physical activity

To assess physical activity, we used the 60 min moderate-to-vigorous physical activity screening measure, demonstrated to be reliable (intraclass correlation, 0.77) and correlate (*r* = 0.40, *p* < 0.001) with accelerometer data [55]. Students were asked to indicate how many days over the past week they were physically active for a total of 60 min or more per day. Responses were binary coded to classify students as engaging in lower or higher levels of physical activity (≤4 days/week cf. ≥5 days/week).

#### Sedentary recreational screen time

Students were asked to record, in hours and minutes, how long they usually spend doing each of the following activities on a usual school day, Saturday, and Sunday: watching TV; watching videos/DVDs; playing on smart phone or tablet (e.g., iPad); playing video games other than on the computer (e.g., Nintendo, Xbox, Playstation); using the computer for fun. Time spent doing each activity was summed and a weighted average daily time spent doing sedentary recreational screen time was calculated, with values dichotomised to indicate students who were exceeding national recreational screen time guidelines (i.e., >2 h/day) [56].

#### Estimated sleep duration

To assess sleep duration, students were asked to record what time they usually go to bed and turn the lights out on a school night and what time they usually wake up on a school day. The difference between students’ wake-up time and bedtime was calculated to estimate usual sleep duration on a school night. A similar approach using single items has been used elsewhere to examine adolescent total sleep time [57], with previous research suggesting there is validity between such self-report measures and both sleep diary and actigraphy in adolescents, particularly for school nights [58]. Students’ estimated sleep duration was then dichotomised to indicate students who were not sleeping for the recommended minimum hours on a typical school night as per the Australian 24 Hour Movement Guidelines for Children and Young People [56]. For students aged up to 13 years, the minimum is 9 h, while for students aged 14–17 years the minimum is 8 h [56].

### Data analysis

Data were analysed using Stata/MP V.16.0 [59]. The analysis was pre-specified, in line with the a priori research question. Specifically, to examine associations (reported as odds ratios [OR]) between regular energy drink consumption and sociodemographic and behavioural factors, we used a single, mutually-adjusted, multilevel logistic regression model with a random effect for school to adjust standard errors for correlations within and between schools. All independent variables were entered simultaneously given previous research indicating that among adolescents many of the examined dietary and lifestyle behaviours have been shown to be associated with each other (e.g., [52–54, 60]).The model also adjusted for state/territory and education sector (government, Catholic and independent). Variance inflation factor indicated there were no issues with multicollinearity between independent variables. Students with missing energy drink consumption or sociodemographic data were excluded from analysis, while all other missing values were treated listwise. Analysis was conducted using a Bonferroni-adjusted alpha level of 0.004 (0.05/14) to account for multiple comparisons.

## Results

### Sample characteristics

Nationally, 104 secondary schools participated in the 2018 NaSSDA survey (school response rate = 8%), with 9102 students in year levels 8 to 11 surveyed in total (student response rate = 67%). This paper reports on data for 8942 students aged 12 to 17 years (M = 14.7, SD = 1.2) who reported their energy drink consumption along with complete sociodemographic information. Of the students included in the final sample, 48% were male. A lower proportion of students were in Year 11 (18%) compared with younger year levels (Year 8: 28%; Year 9: 30%; Year 10: 24%). Just over a quarter of students (29%) were classified as residing in a low socio-economic area and 25% in a high socio-economic area (compared with 34% low and 25% high among the Australian population) [48]. Around two-thirds of students (65%) resided in a metropolitan location (compared with 71% of the Australian population) [61]. Just over a third (36%) of students indicated they had less than AUD$10 weekly spending money, 35% had between AUD$10 and AUD$39, and 29% had at least AUD$40 available to spend on themselves during a typical week.

### Prevalence of energy drink consumption

Overall, 8% (95% CI: 7.3–8.4) of students surveyed were classified as regular energy drink consumers (≥1 cup/week). A further 16% (95% CI: 15.2–16.7) of students reported consuming less than one cup per week of energy drinks, while 76% (95% CI: 75.3–77.1) reported they did not consume these types of drinks.

### Sociodemographic characteristics

After adjusting for all covariates, state/territory and school type, and accounting for a random effect for school, we found evidence of independent associations for two of the sociodemographic factors assessed. As shown in Table 1, males were significantly more likely than females to regularly consume energy drinks, while regular energy drink consumption was significantly more common among students who had at least AUD$40 per week discretionary income compared to those who had less than AUD$10 weekly spending money. Regular energy drink consumption was not found to be significantly associated with year level, socio-economic area, or geographic location.

**Table 1:** Results from a multilevel logistic regression model examining demographic characteristics and health behaviours associated with energy drink consumption among Australian secondary school students (*N* = 8736)

	Energy drink consumption (≥1 cup/week)			
	Adjusted %	Adjusted OR	95% CI	*P* value
**Demographic characteristics**				
* Sex*				
Female	5.2	1.00		
Male	8.3	**1.83**	**1.49–2.24**	**<0.001**
* Year level*				
8	6.1	1.00		
9	6.7	1.13	0.87–1.47	0.367
10	8.1	1.43	1.09–1.89	0.011
11	6.6	1.11	0.80–1.53	0.538
*Socio-economic area*				
High (least disadvantaged)	6.3	1.00		
Medium	7.1	1.17	0.87–1.57	0.300
Low (most disadvantaged)	7.1	1.16	0.83–1.63	0.386
*Geographic location*				
Metropolitan	6.9	1.00		
Rural/regional	7.0	1.03	0.72–1.47	0.873
*Weekly spending money*				
<AUD$10	5.2	1.00		
AUD$10–AUD$39	6.6	1.35	1.06–1.72	0.014
≥AUD$40	**9.0**	**2.03**	**1.60–2.57**	**<0.001**
**Health behaviours**				
* Vegetable consumption*				
≥2 servings/day	6.6	1.00		
≤1 serving/day	7.7	1.22	1.00–1.50	0.055
* Fruit consumption*				
≥2 servings/day	7.3	1.00		
≤1 serving/day	6.0	0.76	0.62–0.95	0.015
* Fruit juice consumption*				
≤3 cups/week	6.1	1.00		
≥4 cups/week	**8.6**	**1.57**	**1.29–1.90**	**<0.001**
* Sugary drink consumption*				
≤3 cups/week	4.5	1.00		
≥4 cups/week	**13.7**	**3.81**	**3.12–4.66**	**<0.001**
* Fast food consumption*				
<1 times/week	4.9	1.00		
≥1 times/week	**8.6**	**2.00**	**1.62–2.45**	**<0.001**
* Snack food consumption*				
<14 times/week	5.9	1.00		
≥14 times/week	**10.4**	**2.08**	**1.69–2.56**	**<0.001**
* Physical activity*				
≤4 days/week	6.9	1.00		
≥5 days/week	6.9	1.00	0.83–1.21	0.981
* Sedentary recreational screen time*				
≤2 h/day	7.2	1.00		
>2 h/day	6.9	0.94	0.65–1.36	0.739
* Estimated sleep duration*^a^				
Meeting recommendations	5.5	1.00		
Not meeting recommendations	**9.8**	**2.10**	**1.74–2.53**	**<0.001**

Notes: Bolded results are statistically significant at *p* < 0.01. Percentages and odds ratios are adjusted for all other covariates listed in the table, school-level clustering, state/territory and school type

*OR* odds ratio, *CI* confidence interval, *TV* television

^a^For students aged 5 to 13 years, the recommended minimum sleep is 9 h per night, while for students aged 14 to 17 years, the recommended minimum sleep is 8 h per night [56]

### Health behaviours

As presented in Table 1, the odds of consuming energy drinks at least weekly was independently associated with five behavioural characteristics, including four of the six diet-related behaviours examined. Specifically, the factor most strongly associated with regular energy drink consumption was consumption of other sugary drinks, with students who reported drinking four or more cups of other varieties of sugary drinks in a usual week being nearly four times as likely to also regularly consume energy drinks compared to students who usually drank three or less cups of other sugary drinks. Regular energy drink consumption was also more common among students who reported consuming four or more cups of fruit juice in a usual week. Students were around twice as likely to regularly consume energy drinks if they reported eating snack foods 14 or more times per week or fast food at least weekly. Regular energy drink consumption was not found to be significantly related to daily intake of vegetables or fruit. Students’ physical activity and sedentary recreational screen time were also not significantly associated with regular energy drink consumption. However, students who were not meeting sleep recommendations were more than twice as likely to be regular energy drink consumers compared to those who reported sleeping for at least the minimum recommended hours on a usual school night.

## Discussion

To our knowledge, this is the first Australian study to employ a large, national sample to examine patterns of energy drink consumption in adolescents, finding that around one in 13 students (8%) reported consuming at least one cup of energy drinks in a usual week. This figure is similar to a recent US study using a nationally representative sample which found 8% of 13- to 17-year-olds consumed energy drinks weekly [29]. Other studies have reported between 10 and 19% weekly use among adolescents [33, 39, 45, 62–66], while some have produced much higher estimates ranging between a quarter to over a third of adolescents consuming energy drinks weekly [32, 67, 68]. These differences may reflect methodological approaches, such as varying referent periods to assess consumption (e.g., usual cf. last 7 days). Alternatively, they may reflect cultural or geographical differences in energy drink consumption, with studies showing, for example, substantial variability in adolescents’ consumption across Europe [69] and higher intake in Northern compared to Southern England [67]. While the majority of adolescents in the current study did not consume energy drinks, there was evidence that regular consumption was more prevalent among males and students with greater weekly spending money. Regular consumption also clustered with other unhealthy dietary behaviours. Importantly, it was also associated with short sleep duration.

Consistent with previous studies in Australia [62] and elsewhere [9, 25, 32, 44, 67, 70], we found regular energy drink consumption was significantly higher among males at 8%. This is unsurprising given marketing for these products is heavily directed towards males, such as through sports and video games [20, 21, 71], and the gender difference in consumption is also observed in adults [72]. However, since 5% of female students were regular consumers, strategies to reduce consumption among all adolescents are needed, given this age group is particularly susceptible to the potential harms associated with energy drink consumption [17]. In addition, adolescence is a period of increasing independence in food purchasing and consumption [73] and represents a critical juncture during which lifetime eating behaviours may be established [73, 74]. This increasing autonomy is reflected in our finding that energy drink consumption was associated with greater weekly spending money, an observation reported previously by others [31, 38].

Our findings indicated that regular energy drink consumption did not vary by year level, geographic location, or socio-economic area. Previous research examining energy drink consumption by year level or age has yielded inconsistent results [44], with some studies finding consumption increased with year level or age [41, 62–64], while others observed an inverse association [24, 26, 43], differing effects by gender [38], or no association [9, 68]. These conflicting findings may be due to how energy drink consumption is classified across studies (i.e., any cf. weekly cf. daily consumption). Similarly, the few studies that have examined adolescents’ energy drink consumption as a function of geographic location or socio-economic factors have generated mixed findings. For example, a study using a sample of Swiss adolescents found energy drink consumption was higher among those living in urban areas [25], while one US study reported past 24-h energy drink use was lower among adolescents from low-income families [75], and another found consumption was lower among those with more educated parents and higher among those living outside of metropolitan locations [28]. Data from the UK indicated weekly consumption was more prevalent among students eligible for welfare (i.e., free school meals) [41, 67], and some research has not found a significant association between consumption and socio-economic area [26]. Further research would be helpful to explicate the relationship between energy drink use and socio-economic and geographic factors, with examination of both area- and individual-level measures likely to provide greatest insight.

In line with previous research [32, 39–41, 76], weekly energy drink consumption was associated with higher intake of other sugary drinks, fruit juice, fast food and snack foods. This clustering of unhealthy eating behaviours suggests that interventions aimed at improving the whole diet rather than focusing on energy drinks alone may have greatest impact in improving adolescents’ health and other outcomes. Indeed, a recent UK study showed that energy drink consumption was inversely related to academic achievement, but the significant effect was diminished when another unhealthy diet variable (i.e., junk food) was added to the model, indicating that energy drinks should be considered as part of overall diet [76].

We found that students who were not sleeping for the recommended minimum hours on a typical school night were more likely to regularly consume energy drinks. This aligns with previous research showing an inverse association between adolescents’ energy drink consumption and sleep duration [25, 41, 46] and converges with studies reporting positive associations between energy drink use and various sleeping problems [44, 45]. Although the direction of causation cannot be established from the present cross-sectional data, previous research suggests that this association may in part be due to adolescents using energy drinks to counteract sleep deficits [77]. Unfortunately, a range of biological and environmental factors predispose adolescents to compromised sleep [78]. Further studies are needed to determine the extent to which addressing adolescents’ sleep behaviours may reduce their consumption of energy drinks.

### Study implications

The current results identify key target intervention groups and modifiable behaviours that can be addressed alongside energy drink consumption. Effective interventions will likely require both environmental- and individual-level approaches. Public health advocates in Australia have called for age restrictions on the sale of energy drinks to children and adolescents (as has occurred in some European countries) [79–81] and, given the strong evidence linking unhealthy food advertising with enhanced attitudes, preferences for and consumption of marketed foods [82, 83], restrictions on the advertising and marketing of unhealthy food and beverages to children and adolescents generally [84, 85]. A health levy on sugary drinks has also been recommended [86], with evidence from countries where such taxes have been implemented demonstrating it to be an effective avenue to both reduce sugary drink consumption and encourage manufacturers to reduce the sugar content of drink products [87, 88]. Notably, due to their sensitivity to price, young people are among those most likely to respond to such taxes and therefore are expected to benefit the most in terms of related health gains [89]. As part of a comprehensive strategy, other complementary environmental interventions may include policies to reduce availability of sugary drinks in public settings, such as schools [90], hospitals [91], and community recreation venues [92].

In addition to effective regulatory measures, media literacy and counter-advertising strategies that alert adolescents to the persuasive tactics used by the unhealthy food industry, including energy drink brands, may serve to diminish the effects of industry marketing. For example, a recent US study found that framing marketing by unhealthy food companies as contradicting youth values, such as social justice and autonomy, decreased students’ implicit positive associations with junk food marketing and, among male students, improved daily school food choices [93]. Given the need to develop strategies appropriate for male adolescents, this approach may be promising in reducing energy drink consumption, as well as improving other dietary behaviours.

Raising adolescents’ awareness about the detrimental effects of energy drinks may also prove beneficial, with previous research finding that possessing such knowledge served as a barrier to consumption [9, 20, 70]. Currently among Australian adolescents there is a lack of awareness of energy drink ingredients and their actions [20, 94], as well as confusion about serving size and recommended daily limit [20, 62]. Implementing standards for efficacious labelling may be one way to improve knowledge [20, 94, 95], and is a strategy that could be applied to unhealthy foods and beverages more broadly. Similarly, interventions targeting parents may offer a promising avenue for reducing energy drink consumption, as parents have been identified as facilitating consumption through role modelling and providing energy drinks to their adolescents [20, 94], as well as deterring consumption through expressing disapproval of these products [20, 27].

### Strengths and limitations

Strengths of the current study include the large, national sample and the ability to quantify both the frequency and amount of energy drinks Australian adolescents are consuming, whereas most other studies have typically only captured frequency of consumption. Several limitations, however, should be considered. First, the cross-sectional design precludes inferences of causality. Second, outcomes were self-reported and may be subject to recall or social desirability bias [96]. Additionally, due to survey constraints, energy drink consumption was assessed with a single item question about *usual* consumption with no specified referent period and categorical responses, which may be subject to measurement error; however, *usual* intake is generally most relevant from a public health perspective [97]. Students’ total caffeine intake, including from sources other than energy drinks such as caffeinated soft drinks, tea, coffee and caffeinated pills, was not captured and may be an important confounder when examining the association between energy drink consumption and sleep variables and represents an avenue for future research. Reflecting the challenges of implementing surveys in school settings, the response rate achieved was low, though the selection of schools with similar characteristics (i.e., state, sector, postcode) to replace refusals aided in conserving the representativeness of the sample. Finally, we were unable to include weight status (based on BMI category) in our statistical model due to missing self-reported height and weight data diminishing the sample size extensively. Although previous studies have produced mixed results regarding weight status and energy drink consumption [38, 39, 47, 66, 98] it may be an important confounder, and future studies should seek to include this variable where possible.

## Conclusions

The current results indicate that Australian adolescents’ energy drink consumption is most prevalent among males and typically occurs amidst a cluster of unhealthy dietary behaviours and short sleep duration. Findings support advocacy efforts for policies that will reach identified at-risk groups, such as increased regulation of the marketing and sale of energy drinks, as well as highlight opportunities for interventions targeting energy drinks alongside other unhealthy dietary behaviours.

## Data Availability

The dataset supporting the conclusions of this article and a copy of the survey tool are available from the corresponding author upon reasonable request.

## Abbreviations

ASGS: Australian Statistical Geography Standard
BMI: Body mass index
IRSD: Index of Relative Socio-Economic Disadvantage
NaSSDA: National Secondary Students’ Diet and Activity
NNPAS: National Nutrition and Physical Activity Survey
NSW: New South Wales
SPANS: Schools Physical Activity and Nutrition Survey
